# PD-L1 expression levels in mesenchymal stromal cells predict their therapeutic values for autoimmune hepatitis

**DOI:** 10.1186/s13287-023-03594-z

**Published:** 2023-12-18

**Authors:** Xilong Bai, Tingwei Chen, Yuqi Li, Xiaofan Ge, Caie Qiu, Huili Gou, Sili Wei, Tingting Liu, Wei Yang, Liting Yang, Yingmin Liang, Zhansheng Jia, Liangshan Lv, Tianqing Li

**Affiliations:** 1 State Key Laboratory of Primate Biomedical Research, Institute of Primate Translational Medicine, Kunming University of Science and Technology, Kunming, 650500 Yunnan China; 2 Xi’an ChaoYue Stem Cell Co., Ltd, Xi’an, 710100 Shaanxi China; 3 Department of Hematology, Xi’an International Medical Center Hospital, Xi’an, 710100 Shaanxi China; 4 Department of Infection and Liver Disease, Xi’an International Medical Center Hospital, Xi’an, 710100 Shaanxi China; 5 Department of Minimally Invasive Interventional Radiology, Xi’an Gaoxin Hospital, Xi’an, , 710075 Shaanxi China

**Keywords:** Mesenchymal stromal cells, Cellular heterogeneity, PD-L1, Immunoregulation, Autoimmune hepatitis

## Abstract

**Background:**

Autoimmune hepatitis is a chronic inflammatory hepatic disorder with no effective treatment. Mesenchymal stromal cells (MSCs) have emerged as a promising treatment owing to their unique advantages. However, their heterogeneity is hampering use in clinical applications.

**Methods:**

Wharton’s jelly derived MSCs (WJ-MSCs) were isolated from 58 human donors using current good manufacturing practice conditions. Gene expression profiles of the WJ-MSCs were analyzed by transcriptome and single-cell RNA-sequencing (scRNA-seq), and subsequent functional differences were assessed. Expression levels of programmed death-ligand 1 (PD-L1) were used as an indicator to screen WJ-MSCs with varied immunomodulation activities and assessed their corresponding therapeutic effects in a mouse model of concanavalin A-induced autoimmune hepatitis.

**Results:**

The 58 different donor-derived WJ-MSCs were grouped into six gene expression profile clusters. The gene in different clusters displayed obvious variations in cell proliferation, differentiation bias, trophic factor secretion, and immunoregulation. Data of scRNA-seq revealed four distinct WJ-MSCs subpopulations. Notably, the different immunosuppression capacities of WJ-MSCs were positively correlated with PD-L1 expression. WJ-MSCs with high expression of PD-L1 were therapeutically superior to WJ-MSCs with low PD-L1 expression in treating autoimmune hepatitis.

**Conclusion:**

PD-L1 expression levels of WJ-MSCs could be regarded as an indicator to choose optimal MSCs for treating autoimmune disease. These findings provided novel insights into the quality control of MSCs and will inform improvements in the therapeutic benefits of MSCs.

**Supplementary Information:**

The online version contains supplementary material available at 10.1186/s13287-023-03594-z.

## Background

Autoimmune hepatitis (AIH) is one of the chronic immune-mediated liver disease, which is characterized by elevated levels of liver transferases and IgG, positive serum autoantibodies, and interfacial inflammation of liver tissue [1]. The prevalence of AIH is 17.44 per 100,000 people in a global context [2]. AIH affects people of all ages, but occurs more often in women [3]. The pathogenesis of AIH is considered to involve multiple types of immune cells that are recruited into the liver in response to the production of self-antigens, leading to an inflammatory immune reaction [4]. Importantly, long-term chronic inflammation in the liver leads to hepatic fibrosis, which can ultimately progress to end-stage liver diseases, such as liver cirrhosis and liver failure, and even hepatocellular carcinoma [5]. At present, corticosteroids are the common treatment for AIH, but there are many problems with this treatment. For instance, some patients experience severe adverse effects after treatment or recurrence following discontinued steroid use after recovery [6]. Thus, a new therapy for treating AIH patients is urgently needed.

Mesenchymal stromal/stem cell (MSC)-based therapy is a promising therapeutic strategy for AIH due to the special properties and functions of MSCs [7]. However, in different clinic assays, the therapeutic benefits of MSCs are quite varied. An important reason for this variability is the heterogeneity of MSCs, which leads to the inability to obtain consistent MSCs and ultimately hampers clinical benefits [8]. Therefore, it is necessary to screen optimal MSCs in a disease-specific manner to ensure clinical efficacies [9].

The pathogenesis of AIH indicates that screening MSCs with strong immunomodulatory ability using specific indicators or cell surface markers may be very important for the treatment of AIH. Evidence indicates that the immunosuppressive properties of MSCs are mediated by direct or indirect cell-to-cell communication to educate immune cells and ultimately regulate the disease-specific microenvironment [10]. Cell-to-cell direct communications are mediated by various surface molecules, including C-X-C motif chemokine receptor 4, programmed death-ligand 1 (PD-L1), and inter-cellular adhesion molecule 1 [11–13]. Cell-to-cell indirect communication is achieved through soluble factors secreted by MSCs, including indoleamine 2,3-dioxygenase, prostaglandin E2, PD-L1, or tumor necrosis factor-stimulated gene-6 [14–17]. Both direct and indirect pathways are critical for immunosuppression of MSCs. Notably, PD-L1 as a cell surface molecule could also be secreted extracellularly.

PD-L1, also known as CD274, is vital for the inhibition of inflammation. The discovery that PD-L1 is highly expressed on MSCs spurred research on the roles of PD-L1 [18]. Many studies have established that the immunosuppressive properties of MSCs on T cells are mediated by targeting programmed cell death protein 1 (PD-1)/PD-L1. Although it is unclear whether PD-L1 can involve in the therapeutic benefits of MSCs on AIH, blockage of PD-L1 signaling on MSCs significantly eliminates the immunosuppressive capacities of MSCs [11]. Therefore, we propose that MSCs that highly express PD-L1 may be optimal MSCs for treating AIH.

In this study, we revealed the variations of genes related to cell proliferation, differentiation bias, trophic factor secretion, and immune function in human Wharton’s jelly derived MSCs (WJ-MSCs) from 58 different donors by analyzing transcriptome and single-cell RNA sequencing (scRNA-seq). We also examined differences of WJ-MSCs from different donors concerning differentiation bias and immunoregulation ability. Different donor-derived WJ-MSCs exhibited remarkable varieties in immunosuppression, which were positively correlated with their expression levels of PD-L1. Importantly, using a mouse model of concanavalin A (ConA)-induced autoimmune hepatitis, we revealed that WJ-MSCs with high expression of PD-L1 (PD-L1^high^ WJ-MSCs) produced better therapeutic efficacy and stronger immunosuppression compared to PD-L1^low^ WJ-MSCs by altering T subset proliferation and reducing inflammation.

## Materials and methods

This research was approved by the Ethics Committee of Xi'an Gaoxin Hospital (GXYYEC-KTSB-2020-03-01) and the Institutional Animal Care and Use Committee of Kunming University of Science and Technology (PZWH-K2022-0014). All umbilical cord and peripheral blood mononuclear cells (PBMCs) samples were taken after informed and written consent. Our reporting adheres to the ARRIVE guidelines.

### Cell isolation and culture

MSCs were manufactured in clean environments in accordance with requirements of current good manufacturing practice (cGMP). MSCs were isolated from WJ of healthy donors (Table 1). Expanded MSCs were then seeded in T175 flasks and cultured in serum-free medium (YOCON, Beijing, China) at 37 °C with 5% CO_2_ in a humidified atmosphere. After the cell density reached approximately 80% confluence, cells were dissociated with TrypLE™ (Gibco, Billings, MT, USA) at 37 °C for 5 min. MSCs at the third passage were harvested and immediately used for scRNA library construction, cell surface marker staining, or tri-lineage differentiation test, or were frozen in liquid nitrogen for long-term storage.

**Table 1:** Information of WJ-MSCs

Name	Fetal gender	Donor age	Name	Fetal gender	Donor age
WJ-MSCs01	Male	27	WJ-MSCs30	Female	30
WJ-MSCs02	Male	25	WJ-MSCs31	Female	31
WJ-MSCs03	Male	30	WJ-MSCs32	Female	28
WJ-MSCs04	Male	27	WJ-MSCs33	Female	30
WJ-MSCs05	Male	28	WJ-MSCs34	Male	31
WJ-MSCs06	Female	31	WJ-MSCs35	Female	27
WJ-MSCs07	Male	30	WJ-MSCs36	Male	29
WJ-MSCs08	Female	29	WJ-MSCs37	Female	28
WJ-MSCs09	Male	26	WJ-MSCs38	Female	26
WJ-MSCs10	Male	31	WJ-MSCs39	Male	28
WJ-MSCs11	Female	28	WJ-MSCs40	Female	31
WJ-MSCs12	Male	27	WJ-MSCs41	Male	29
WJ-MSCs13	Male	26	WJ-MSCs42	Male	30
WJ-MSCs14	Male	27	WJ-MSCs43	Male	29
WJ-MSCs15	Male	30	WJ-MSCs44	Female	31
WJ-MSCs16	Male	26	WJ-MSCs45	Female	30
WJ-MSCs17	Female	25	WJ-MSCs46	Female	30
WJ-MSCs18	Male	28	WJ-MSCs47	Male	25
WJ-MSCs19	Male	31	WJ-MSCs48	Male	25
WJ-MSCs20	Male	28	WJ-MSCs49	Male	31
WJ-MSCs21	Male	29	WJ-MSCs50	Male	28
WJ-MSCs22	Male	27	WJ-MSCs51	Female	27
WJ-MSCs23	Male	28	WJ-MSCs52	Male	26
WJ-MSCs24	Female	29	WJ-MSCs53	Female	29
WJ-MSCs25	Female	26	WJ-MSCs54	Male	30
WJ-MSCs26	Female	28	WJ-MSCs55	Male	27
WJ-MSCs27	Female	30	WJ-MSCs56	Female	28
WJ-MSCs28	Female	27	WJ-MSCs57	Male	24
WJ-MSCs29	Male	28	WJ-MSCs58	Male	27

### Surface marker expression assay

According to guidelines from Mesenchymal and Tissue Stem Cell Committee of the International Society for Cellular Therapy, MSCs have three minimal definition criteria. These are adhesion to plastic, expressions of specific surface markers (CD105, CD73, CD90, positive cells ≥ 95%; CD45, CD34, CD14 or CD11b, CD79a or CD19, and HLA-DR negative cells ≤ 2%), and multilineage differentiation potentials of adipogenesis, osteogenesis and chondrogenesis. To assay surface marker expression, approximately 1 × 10^6^ cells at the third passage were harvested and resuspended in 100 μL PBS, followed by staining with the following monoclonal antibodies labeled with either fluorescein isothiocyanate (FITC) or phycoerythrin (PE): CD34, CD11b, CD45, CD19, CD73, CD105, CD90, and CD44 (BD, San Jose, CA, USA). After incubation in the dark for 30 min at room temperature, cells were washed three times using 1 × PBS and resuspended in washing buffer for flow cytometry analysis using a FACSCanto™ device (BD, San Jose, CA, USA). The data were analyzed with the fluorescence-activated cell sorting software.

### Multi-lineage differentiation assays

For multilineage differentiation, MSCs at the fourth passage were harvested and replated at a density of 1 × 10^4^ cells/well in a 24-well culture plate. When the cells reached 50 ~ 70% confluency, adipogenic and osteogenic media (Gibco, Billings, MT, USA) were replaced to induce adipogenesis and osteogenesis, respectively. After 21 days, cells were fixed in 4% formaldehyde and stained with Oil Red O (Sigma-Aldrich, St, Louis, MO, USA) or Alizarin Red S (Sigma-Aldrich, St, Louis, MO, USA) to evaluate the adipogenic or osteogenic differentiations, respectively. For chondrogenic differentiation, 2 × 10^5^ WJ-MSCs at the fourth passage were centrifuged for 5 min at 1200 rpm in a tube. The supernatant was removed, and the pellet resuspended in chondrogenic medium (Gibco, Billings, MT, USA). After 21 days, the pellet was fixed in 4% formaldehyde, dehydrated through serial ethanol concentrations, and embedded in optimal cutting temperature compound. Blocks were cut into 5-mm-thick sections and stained with Alcian Blue (Sigma-Aldrich, St, Louis, MO, USA).

### Soft agar assay

Agarose solution (1.2%) and 2 × medium were mixed in a ratio of 1:1. A 1.5 mL volume of the mixed medium was quickly added to each well of a 6-well plate, and the gel was allowed to solidify at room temperature. A suspension of 10,000 WJ-MSCs was added to the mixture of 0.7% agarose and 2 × medium (1:1) per well. The mixture was quickly added to 6-well plate at 1 mL per well after mixing. After the upper agar was solidified, the plate was placed in 37 °C incubator in a 5% CO_2_ atmosphere for 2 to 3 weeks. Each cell line was analyzed in triplicate.

### RNA-seq

Total RNA was extracted from MSCs using RNAiso (Takara Bio, Shiga, Japan), and reverse transcription was performed using RNA PCR Kit (Takara Bio, Shiga, Japan). Agarose gel electrophoresis was used for integrity and quality testing of total RNA. Then, 1–2 μg of total RNA was used to construct sequencing libraries using the KAPA Stranded RNA-Seq Library Prep Kit (Illumina, Hayward, CA, USA) according to the manufacturer’s instructions. The mixed sample sequencing library was transformed by NaOH to generate single-stranded DNA and diluted to a concentration of 8 pM and then, amplified by the TruSeq SR Cluster Kit (Illumina, Hayward, CA, USA). The ends of the generated fragments were sequenced 150 cycles using a HiSeq 4000 device (Illumina, Hayward, CA, USA).

### Single-cell library preparation and sequencing

After three passages in culture, MSCs were processed for scRNA-seq on the Chromium platform (10 × Genomics, Pleasanton, CA, USA). Each sample was processed in a single lane on the Chromium instrument, with a targeted cell recovery of 10,000 cells per sample. The scRNA-seq libraries were prepared with the Chromium Single-Cell 3′ Reagent Kit (10 × Genomics, Pleasanton, CA, USA), according to manufacturer’s instructions. Libraries were pooled and sequenced on the Illumina NextSeq 500 in paired-end configuration to a read depth of approximately 24,000 paired-end reads per cell.

### The scRNA-seq data analysis

Processed gene-cell matrices were analyzed in the R statistical environment using the Seurat package. Data were filtered to exclude genes detected in < 3 cells (per tissue source) to exclude cells with < 2500 unique molecular identifiers (UMIs) or > 15,000 UMIs (putative doublets), and to exclude cells with > 3% UMIs assigned to mitochondrial genes (putative dead or dying cells). Gene-cell count matrices were independently normalized with SCTransform. The top 3000 most variable genes (variance-stabilizing transformation) were selected for dimensional reduction by principal component analysis (PCA). Seurat’s Cell Cycle Scoring function was used to score and identify cell cycle phase (G1, G2M, or S) of individual cells. During normalization, cell cycle score differences (G2/M-S) and mitochondrial transcript percentages were included as factors for regression. Data annotated with corresponding clusters were visualized by Uniform manifold approximation and projection. Differentially expressed gene analyses were conducted using edgeR, with additional modifications for scRNA-seq data. Gene expression linear models included factors for cellular gene detection rate, cluster, and cell cycle score differences. Specific contrasts are detailed in relevant Results sections and/or figures. For all differential gene expression testing analyses, genes expressed in < 25% of cells for at least one cluster/group within a contrast were excluded from differential expression results. Differential gene expression tables were further filtered to only include genes with an adjusted *P* < 0.05. Gene Ontology (GO) enrichment analysis was conducted with the goanna function. Biological process GO terms with *P* < 0.0005 were reported in results.

### Immunosuppression assay of peripheral blood mononuclear cells (PBMCs)

WJ-MSCs were plated into 6-well plates (1 × 10^5^/well) and were incubated for 24 h with serum-free medium. Human PBMCs from healthy donor were cultured on top of the MSCs (MSCs/PBMCs ratio, 1:10) in the presence of 1 × 10^5^ Dynabeads Human T-Activator CD3/CD28 (Gibco, Billings, MT, USA) and 30 ng/mL human interleukin-2 (IL-2; PeproTech, Cranbury, NJ, USA). After 3 d of co-culture, the PBMCs were removed and analyzed by flow cytometry. PBMCs were stained with the following antibodies: FITC-conjugated FOXP3 monoclonal antibody (Thermo Fisher Scientific, Agawam, MA, USA), PE-conjugated anti-human CD25 (BD, San Jose, CA, USA), and allophycocyanin (APC)-conjugated anti-human CD4 (BD, San Jose, CA, USA), according to the manufacturer’s instructions. Samples were run on a FACSCanto™ flow cytometer (BD, San Jose, CA, USA). The percentage of proliferating T cells was analyzed by FlowJo version 10. For the PD-L1 blocking assay, 7.5 μg/mL PD-L1 neutralizing antibody (BPS Bioscience, San Diego, CA, USA) was added to the medium with the MSCs during 24 h. The antibodies were removed and washed-off with PBS before the addition of the PBMCs.

### Induction of AIH and cell transplantation

Eight- to 10-week-old BALB/c mice (female) were used for AIH induction by injection of ConA. For ConA-induced acute injury, ConA dissolved in sterile saline (12 mg/kg body weight) was injected intravenously into mice via the tail vein as previously described [19]. Then, 1 × 10^6^ MSCs in a final volume of 200 μL per mouse were administered intravenously within 30 min after ConA. The control group was injected only with PBS. At the end of treatments (24 h after ConA injection), the mice in each group were euthanized for subsequent analysis. Briefly, mice were killed using CO_2_ at 20% chamber replacement rate. Blood samples were collected, and the serum was extracted. Liver tissues were collected for histopathological and biochemical analyses.

### Serum aminotransferase analysis

The levels of aspartate aminotransferase (AST) and alanine aminotransferase (ALT) in the serum were detected using Cobas 4000 analyzer series (Roche, Basel, Switzerland). Enzyme activities are expressed in international units (U/L).

### Histopathology

Collected liver tissues were immediately fixed with 4% paraformaldehyde and embedded in paraffin. Four-micrometer thick sections of tissues were stained with hematoxylin and eosin to evaluate histopathologic damage. The whole slides were scanned digitally with the Panoramic MIDI scanner (3DHISTECH, Budapest, Hungary). The necrotic area of liver tissues was examined by independent pathologists who were blinded to the experiments following a previously published scoring system [20].

### Terminal deoxynucleotidyl transferase dUTP nick end labeling (TUNEL) assay

TUNEL assay was performed using the DeadEnd™ Fluorometric TUNEL System (Promega, Madison, WI, USA). The samples were examined by fluorescence microscopy (Leica, Wetzlar, Germany). The number of positive cells were calculated from observation of five random fields.

### Flow cytometry

Blood was collected from mice (50 μL in each staining tube). Each tube received 1 × Lysis Buffer (Biolegend, San Diego, CA, USA) followed by incubation at 37 °C for 10 to 15 min. The cells were centrifuged at 300 × *g* for 5 min. The supernatant was removed and the cells were stained with peridinin chlorophyll protein complex (PerCP) anti-mouse CD45 antibody, APC anti-mouse CD3 antibody, PE anti-mouse CD8b antibody, FITC anti-mouse CD4 Antibody, True-Nuclear™ One Step Staining Mouse Treg Flow™ Kit (FOXP3 Alexa Fluor® 488/CD25 PE/CD4 PerCP), and FITC anti-mouse IgG1 antibody, PE anti-mouse IgG1 antibody, and APC anti-mouse IgG1 antibody (all from Biolegend, San Diego, CA, USA) as an isotype control. Samples were run on a BD Canto II flow cytometer. The percentage of proliferating T cells was analyzed using FlowJo version 10.

### Statistical analysis

The statistical analysis performed using SPSS IBM 20.0 (IBM, Armonk, New York, USA) or GraphPad Prism version 7.0 (GraphPad Software Inc., La Jolla, California, USA). Data are expressed as mean ± SD, and differences were considered significant at *P* < 0.05. Statistical significance was determined by the t test or ANOVA (^*^P < 0.05, ^**^P < 0.01, ^***^P < 0.001, ^****^P < 0.0001).

## Results

### Different donor-derived WJ-MSCs exhibit transcriptomic variations

**Fig. 1:**
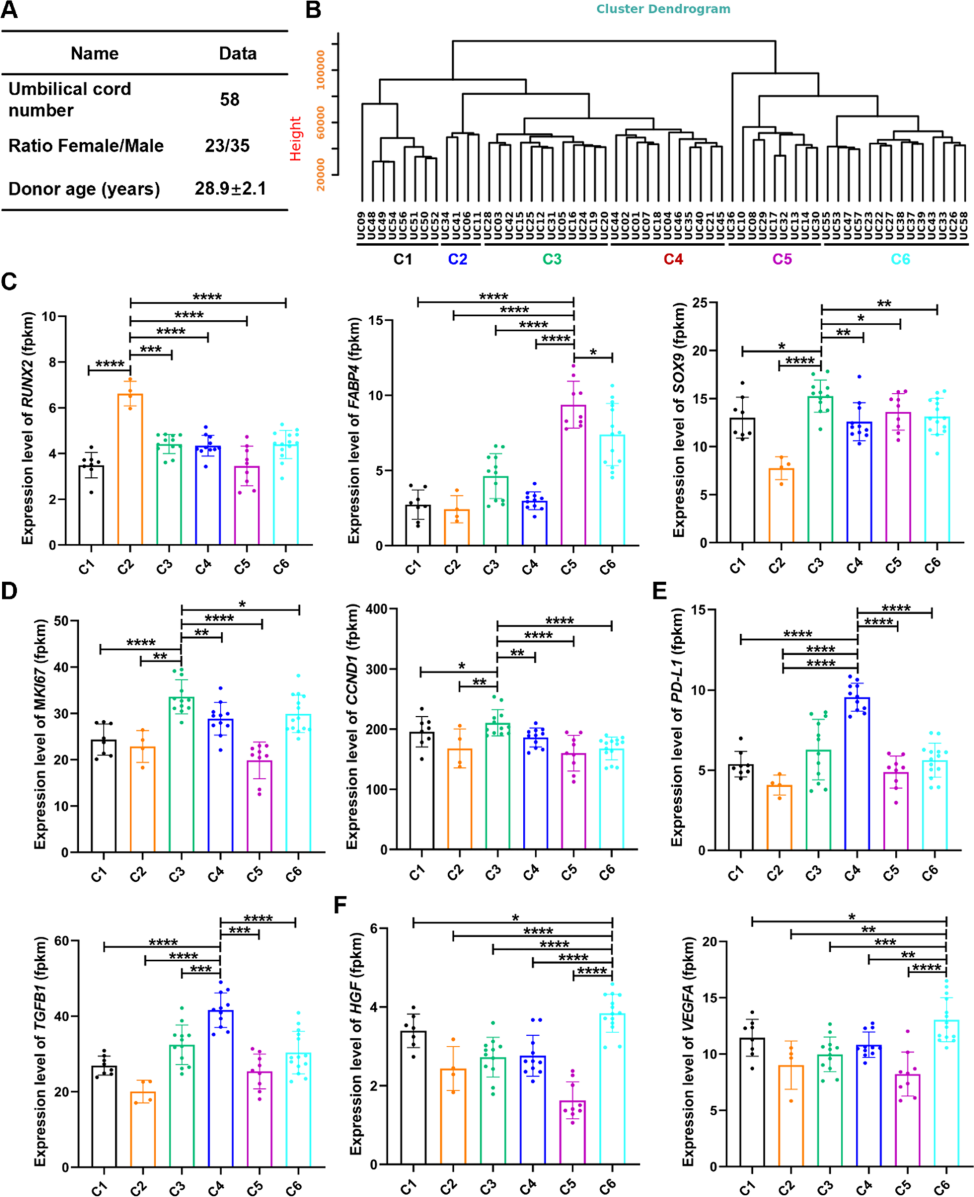
Transcriptomic differences in WJ-MSCs from 58 donors. **A** Sample information from 58 umbilical cords. **B** The evolutionary tree in the form of dendrogram depicting that 58 WJ-MSCs were subgrouped into six different groups. **C** The expression levels of tri-lineage differentiation genes (*RUNX2, FABP4* and *SOX9*) in different WJ-MSCs groups*.* **D** The expression levels of immunoregulatory genes (*PD-L1* and *TGFB1*) in different WJ-MSCs groups. **E** The expression levels of proliferation markers (*MKI67* and *CCND1*) in different WJ-MSCs groups. **F** The expression levels of tissue repair markers (*VEGFA* and *HGF*) in different WJ-MSCs groups. t tests were performed. Statistical significance is indicated by ^*^P < 0.05, ^**^P < 0.01, ^***^P < 0.001, ^****^P < 0.0001

To evaluate the biological differences of MSCs from different donors, 58 different human donor-derived WJ-MSCs were used in this study (Fig. 1A**, **Table 1**)**. These 58 donor-derived WJ-MSCs at passage 5 (P5) were characterized by their differentiation abilities into bone, cartilage, and fat cells (Additional file 1: Fig. S1A), as well as carrying cell surface markers CD44, CD73, CD90, and CD105, but not CD19, CD34, CD45, CD11b, or HLA-DR (Additional file 1: Fig. S1B). The soft agar clone formation test showed no clone formation in all WJ-MSCs at P3, P5, and P10 (Additional file 1: Fig. S1C). However, these WJ-MSCs exhibited variabilities in their cell proliferation abilities (Additional file 1: Fig. S1D). Next, we analyzed the transcriptomes of these 58 different WJ-MSC lines. According to their gene expression profiles, these 58 WJ-MSC lines were grouped into six different clusters in the evolutionary tree cluster analysis (Fig. 1B, Additional file 1: Fig. S1E). Analysis of gene functions of differentially expressed genes among the six groups revealed that different groups exhibited unique molecular characteristics. For lineage differentiation genes, *RUNX2* (osteogenic differentiation gene) and *FABP4* (adipogenic differentiation gene) was highly expressed in Cluster 2, and *SOX9* (chondrogenic differentiation gene) was mainly distributed in Cluster 2, 5, and 3 WJ-MSCs (Fig. 1C). Especially, in Cluster 3, the cell proliferation genes *MKI67* and *CCND1* were activated (Fig. 1D). Cluster 4 was highly enriched for the immune modulation genes, *PD-L1* and *TGFB1* (Fig. 1E). Interestingly, the typical trophic factor genes *VEGFA* and *HGF* were highly concentrated in Cluster 6 (Fig. 1F). The collective observations demonstrated that different donor-derived WJ-MSCs exhibited varied gene expression patterns, which poses huge challenges for benefits of MSCs in treating patients.

### WJ-MSCs include different subpopulations with unique signatures

To uncover the cellular composition and diversity of MSCs, scRNA-seq on WJ-MSCs from three different donors was performed using the high throughput 10 × Genomics platform (Additional file 1: Fig. S2A). Unsupervised clustering was performed after cell cycle regression with the uniform manifold approximation and projection. In total, four clusters were identified (Additional file 1: Fig. S2B). To determine the cellular identity of each cluster, we compared DEGs as well as their corresponding enriched pathways and potential key regulators. Active proliferation was more pronounced in cells in Cluster 1, with high positivity of genes related to DNA replication and cell cycle progression, such as proliferation markers (*TOP2A* and *MKI67*) [21] and the cell cycle regulator *CCDN1* (Additional file 1: Fig. S2C). Interestingly, *CSPG4, MCAM* (CD146), and *NES* [22, 23], which are markers of perivascular mesodermal progenitor cells, and genes essential for maintaining pluripotency and the undifferentiated stem cell state, such as *EZH2* [24], were also highly enriched in Cluster 1 cells (Additional file 1: Fig. S2C). These observations showed that Cluster 1 cells were a potentially stem-like active proliferative cell subpopulation. Cluster 2 cells were enriched with genes for tri-lineage differentiation, including osteogenic differentiation (*RUNX2* and *COL1A1*) [25], adipogenic differentiation (*PPARG* and *FABP4*) [26], and chondrogenic differentiation (*SOX9* and *COL11A1*) [27] (Additional file 1: Fig. S2C). In addition, in Cluster 2 cells, the extracellular matrix remodeling genes *LUM* and *CTSL* were especially activated. These results supported the designation of Cluster 2 cells as a lineage-primed multipotent mesenchymal progenitor cell subpopulation. Cluster 3 cells were enriched with genes crucial for immunomodulation (*PD-L1* and *TGFB1*), indicating that Cluster 3 cells may play roles in immunoregulatory function. The population also highly expressed some genes, such as perivascular mesodermal progenitor cell marker (*NES*), proliferation markers (*TOP2A* and *MKI67*), cycle regulator (*CCND1*), and osteogenic differentiation gene (*COL1A1*) (Additional file 1: Fig. S2C). Cluster 4 cells have some molecular characteristics of pre-smooth muscles with the enrichment with genes essential for smooth muscle contraction (*ACTA2*, *MYL6* and *TPM2*) (Additional file 1: Fig. S2C). Together, these results suggest that WJ-MSCs contained various subpopulations, which might partially explain why different MSCs exhibit functional varieties. The ratios of these subpopulations in MSCs would depend on different donors or isolation and culture methods of WJ-MSCs, which eventually led to produce varied MSCs.

### Different donor-derived MSCs exhibit differentiation bias

**Fig. 2:**
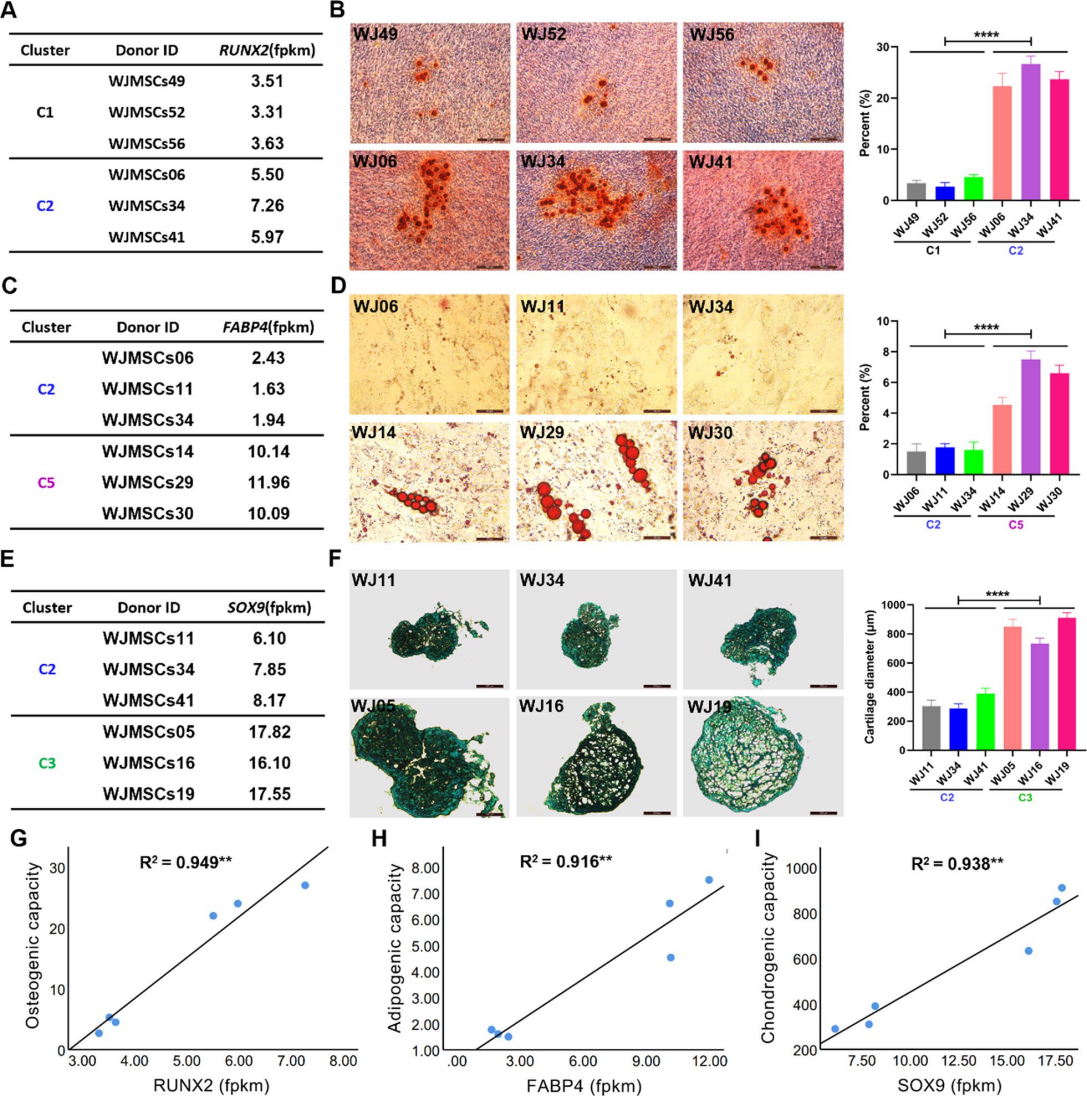
Differences of different donor-derived WJ-MSCs in tri-lineage differentiation bias. **A**, **B** The expression differences of *RUNX2* (fpkm) (**A**) and representative alizarin red staining images of differentiated osteocytes (**B**) in 6 representative WJ-MSCs from the Cluster 1 and 2. The *p* value indicates the significant differences between C1 and C2 groups. **C**, **D** The expression differences of *FABP4* (fpkm) (**C**) and representative oil red O staining images of differentiated adipocytes (**D**) in 6 representative WJ-MSCs from the Cluster 2 and 5. The *p* value indicates the significant differences between C2 and C5 groups. **E**, **F** The expression differences of *SOX9* (fpkm) (**E**) and representative alcian blue staining images of differentiated chondrocytes (**F**) in 6 representative WJ-MSCs from the Cluster 2 and 3. The *p* value indicates the significant differences between C2 and C3 groups. **G**–**I** The correlations between the expression of *RUNX2* in WJ-MSCs and the osteogenic differentiation bias (**G**), the expression of *FABP4* in WJ-MSCs and the adipocyte differentiation bias (**H**), and the expression of *SOX9* in WJ-MSCs and the chondrocyte differentiation bias (**I**). **B**, **D**, **F**, n = 3 each group. t tests were performed. Statistical significance is indicated by ^*^P < 0.05, ^**^P < 0.01, ^***^P < 0.001, ^****^P < 0.0001

Although MSCs differ in their potential to differentiate into osteocytes, adipocytes, and chondrocytes, we observed varieties of differentiation genes among donor-derived MSCs (Fig. 1C). These findings prompted us to speculate that different donor-derived MSCs differ in their bias to become osteoblasts, adipocytes, and chondroblasts. To this end, we selected three different donor-derived *RUNX2*^low^ WJ-MSCs (WJ-MSCs49, WJ-MSCs52 and WJ-MSCs56) in Cluster 1 and three *RUNX2*^high^ WJ-MSCs lines (WJ-MSCs06, WJ-MSCs34 and WJ-MSCs41) in Cluster 2 to investigate osteocyte differentiation potentials (Figs. 1C and 2A). The osteogenic differentiation assay showed that *RUNX2*^high^ WJ-MSCs were inclined to give rise to more osteoblasts than *RUNX2*^low^ WJ-MSCs (Fig. 2B). Similarly, *FABP4*^high^ WJ-MSCs (WJ-MSCs14, WJ-MSCs29 and WJ-MSCs30) in Cluster 5 displayed a stronger adipocyte differentiation ability than *FABP4*^low^ WJ-MSCs (WJ-MSCs06, WJ-MSCs11 and WJ-MSCs34) in Cluster 2 (Fig. 2C, D). Cluster 3 *SOX9*^high^ WJ-MSCs (WJ-MSCs11, WJ-MSCs34 and WJ-MSCs41) were prone to give rise to more chondrocytes than Cluster 2 *SOX9*^low^ WJ-MSCs (WJ-MSCs05, WJ-MSCs16 and WJ-MSCs19) (Fig. 2 E, F). Further correlations revealed that the expression levels of *RUNX2, FABP4*, and *SOX9* in WJ-MSCs appeared to be positively correlated with osteocyte, adipocyte, and chondrocyte differentiation, respectively (Fig. 2G–I). Together, these results showed that biological functions and phenotypes of MSCs could be predicted based on expression levels of individual genes.

### ***PD-L1***^***high***^*** WJ-MSCs have stronger immunomodulatory capacity than PD-L1***^***low***^*** WJ-MSCs***

**Fig. 3:**
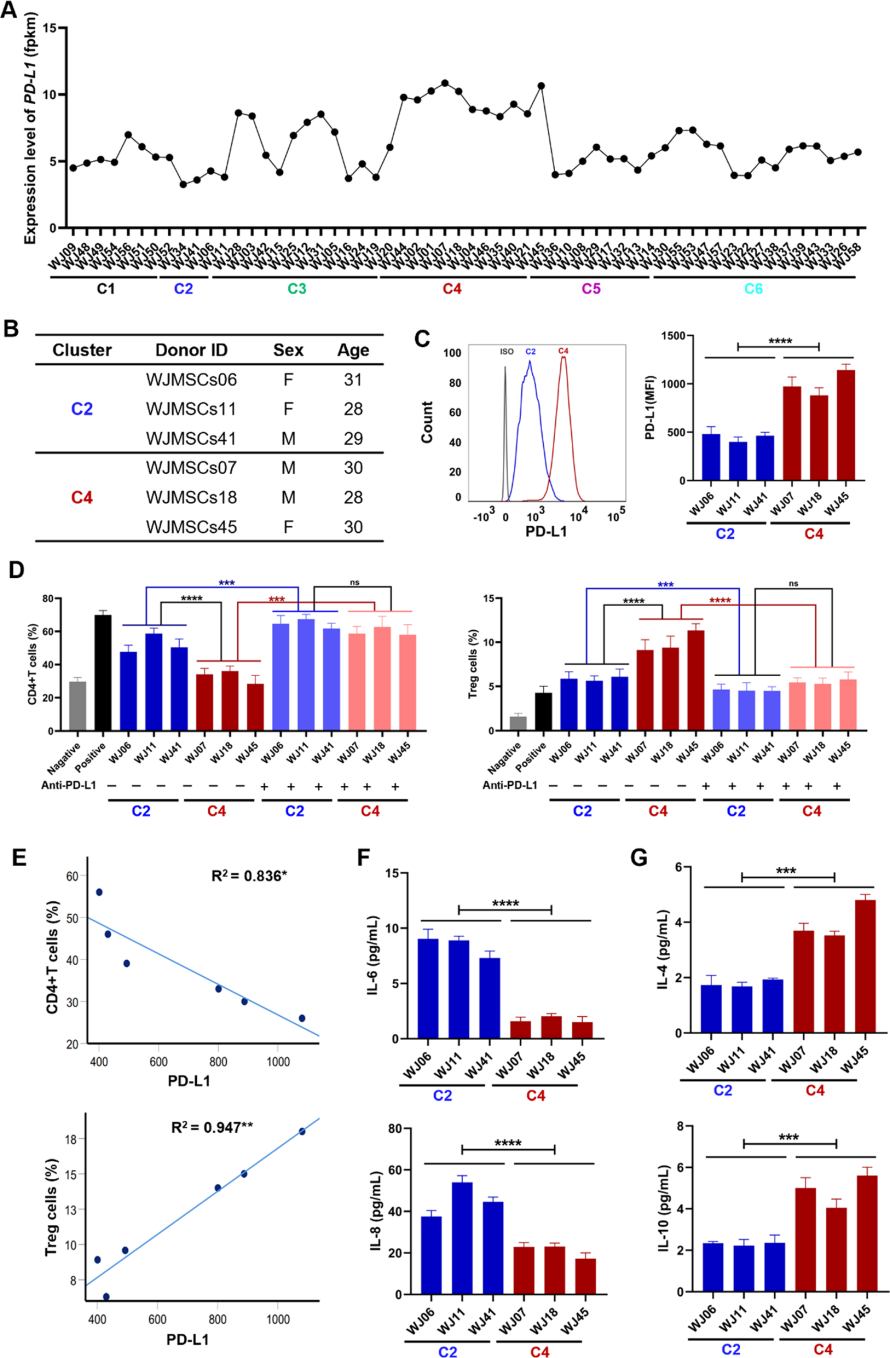
PD-L1^high^ WJ-MSCs have stronger immunomodulatory capacity than PD-L1^low^ WJ-MSCs. **A** Transcriptome analysis of the expression levels of *PD-L1* from 58 WJ-MSCs. **B** Sample information of 6 representative WJ-MSCs from the Cluster 2 and C4. *PD-L1* lowly expressed in WJ-MSCs06, WJ-MSCs11 and WJ-MSCs41, but highly expressed in WJ-MSCs07, WJ-MSCs18 and WJ-MSCs45. **C** Quantification of PD-L1(MFI) expression levels in 6 different WJ-MSCs by flow cytometry. The gray line is isotype control, the blue line is Cluster 2, and the red line is Cluster 4. **D** The effects of PD-L1 neutralizing antibody on WJ-MSCs in inhibiting the proportion of CD4^+^T cells and increasing the production of Treg subpopulation in PBMCs. n = 3 in each group. **E** The correlation analysis between the expression of PD-L1 in WJ-MSCs and the proportions of CD4^+^T cells (**F**) or Treg cells (**G**). F-G. Quantification of pro-inflammatory cytokines (**F**) and anti-inflammatory cytokines (**G**) in the secretomes of WJ-MSCs by ELISA assays. n = 3 in each group. t tests were performed. The *p* value indicates the significant differences between C2 and C4 groups. Statistical significance is indicated by ^*^P < 0.05, ^**^P < 0.01, ^***^P < 0.001, ^****^P < 0.0001

Next, we assessed our proposal that the immunomodulatory capacity of WJ-MSCs could be predicted by single gene expression levels. We observed variable expression of *PD-L1*, which encoded an important immunomodulatory molecule, among 58 the different donor-derived MSCs (Figs. 1E and 3A). We next selected three different donor-derived *PD-L1*^low^ WJ-MSCs (WJ-MSCs06, WJ-MSCs11 and WJ-MSCs41) in Cluster 2 and three *PD-L1*^high^ WJ-MSCs (WJ-MSCs07, WJ-MSCs18 and WJ-MSCs45) in Cluster 4 to assess the relationship between the expression level of *PD-L1* in MSCs and their immunomodulatory capacities (Fig. 3B). We confirmed the differential expression of PD-L1 in these six WJ-MSCs by flow cytometry analysis (Fig. 3C). Because MSCs play a therapeutic role in various immune diseases by regulating immune cells in recipients, we assessed their immunoregulatory effects by examining changes of T cell subpopulations in human PBMCs before and after coculture with each WJ-MSC line. As expected, treatment with WJ-MSCs significantly inhibited the proliferation of CD4^+^T cells and increased the number of the Treg subpopulation in PBMCs (Fig. 3D**, **Additional file 1: Fig. S3). However, blocking PD-L1 by the addition of an anti-PD-L1 antibody after incubation with MSCs before co-culture of MSCs with PBMCs impeded the effects of MSCs on CD4^+^T cell proliferation and Treg cell production (Fig. 3D). Importantly, the expression levels of PD-L1 in WJ-MSCs were positively correlated with the MSC-mediated inhibition of the CD4^+^T cell proliferation and promotion of Treg cell production (Fig. 3E). Furthermore, pro-inflammatory cytokines including IL-6 and IL-8 were lower in PD-L1^high^ WJ-MSCs from Cluster 4 than PD-L1^low^ WJ-MSCs from Cluster 2 (Fig. 3F). The results for anti-inflammatory IL-4 and IL-10 were the opposite (Fig. 3G). These data suggested that the level of expression of PD-L1 could be an indicator to predict the therapeutic values of donor-derived MSCs for human immunomodulatory diseases.

### PD-L1 variations in WJ-MSCs affect therapeutic benefits for autoimmune hepatitis in mice

**Fig. 4:**
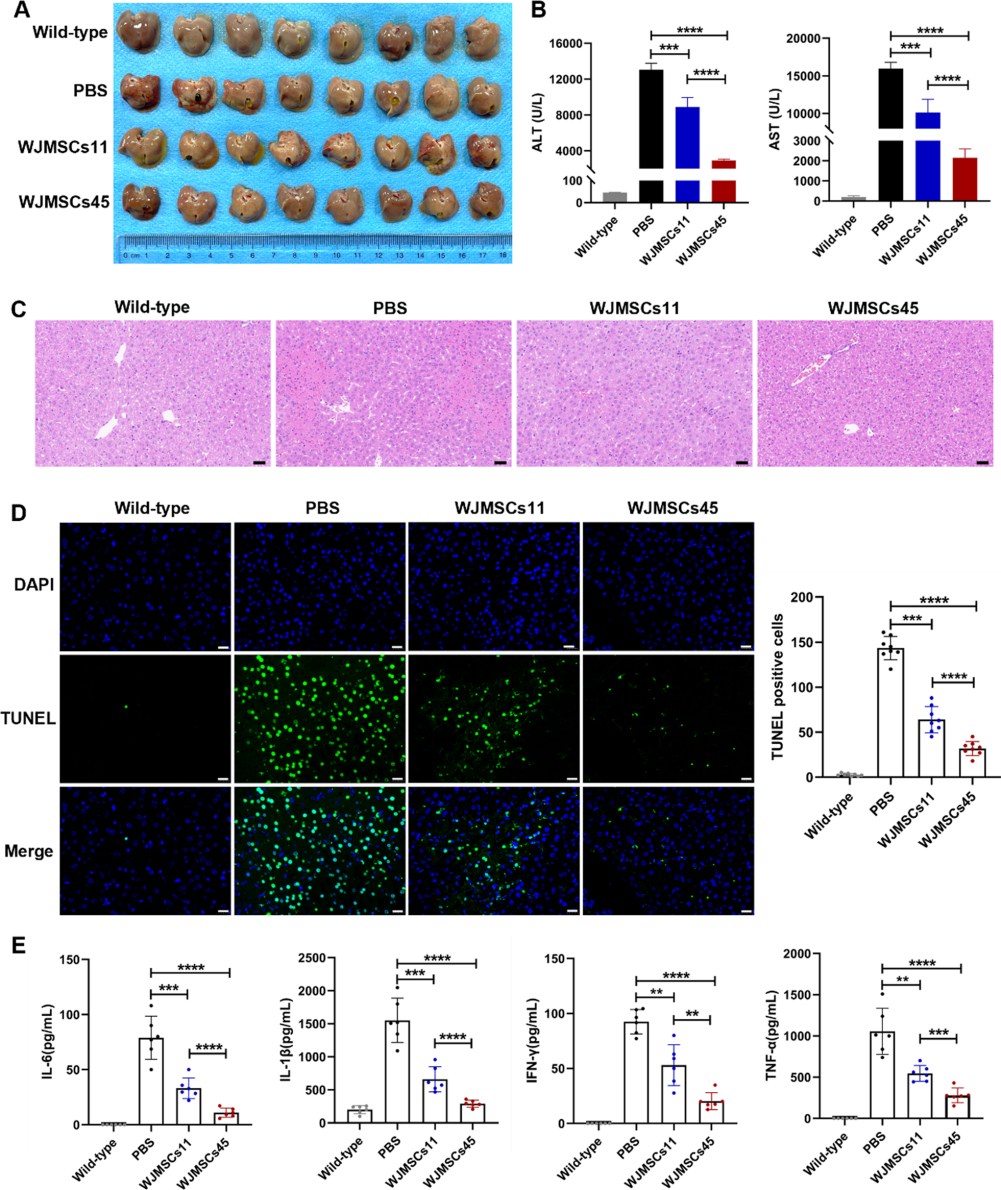
PD-L1 variations in WJ-MSCs to affect therapeutic benefits of for autoimmune hepatitis in mice. **A** Phenotypes of mouse livers in different groups 24 h after MSCs injections. Mice were intravenously injected with 12 mg/kg ConA, followed by intravenous injection with PBS, WJ-MSCs11 or WJ-MSCs45, respectively. n = 8 in each group. **B** Quantification of serum ALT and AST levels. n = 8. **C** Representative H&E staining images of liver histopathology from four different group mice. Scale bar: 100 μm. **D** Quantification of TUNEL-positive cells in four group mice, wild-type, PBS-, WJ-MSCs11-, and WJ-MSCs45- treatment after ConA injection. Scale bar: 20 μm. E. Quantification of IL-6, IL-1β, IFN-γ, and TNF-α in serum from four different group mice by flow cytometry. n = 6 in each group. t tests were performed. Statistical significance is indicated by ^*^P < 0.05, ^**^P < 0.01, ^***^P < 0.001, ^****^P < 0.0001

Among all tested WJ-MSC lines, WJ-MSCs45 most potently inhibited CD4^+^T cell proliferation and increased the Treg subpopulation in PBMCs, while WJ-MSCs11 had limited capability (Fig. 3E). We therefore proposed that the remarkable individual differences between WJ-MSCs in immune regulation could lead to different therapeutic effects in treating diseases. To explore this idea, we chose WJ-MSCs11 and WJ-MSCs45 as representatives to examine their therapeutic efficacy on treating AIH induced in mice. The mouse model of AIH was established by tail vein injection of 12 mg/kg ConA. Mice injected with ConA displayed a series of abnormalities including lethargy, arched back, and hair bristling. The mice with ConA-induced AIH were randomly divided into three groups. The control group were intravenously injected with PBS. The PD-L1^low^ (WJ-MSCs11) and PD-L1^high^ (WJ-MSCs45) groups were individually injected 1 × 10^6^ cells each mice. As expected, WJ-MSCs45 injection produced more effective therapeutic effects than WJ-MSCs11, including improved structure of hepatic lobule, decreased area of liver necrosis, reduced serum ALT and AST, and inhibition of hepatocyte apoptosis (Fig. 4A–D). Compared with WJ-MSCs11, WJ-MSCs45 cells significantly reduced the serum levels of pro-inflammatory factors, IL-6, IL-1β, interferon-gamma, and tumor necrosis factor-alpha (Fig. 4E). Taken together, these data suggested that PD-L1 variations of donor-derived WJ-MSCs were closely related to the therapeutic efficacy for AIH in the mouse model. The findings implicated PD-L1 as a useful indicator in choosing MSCs when clinically treating autoimmune diseases.

### WJ-MSCs attenuate ConA-induced autoimmune hepatitis by regulating T cells

**Fig. 5:**
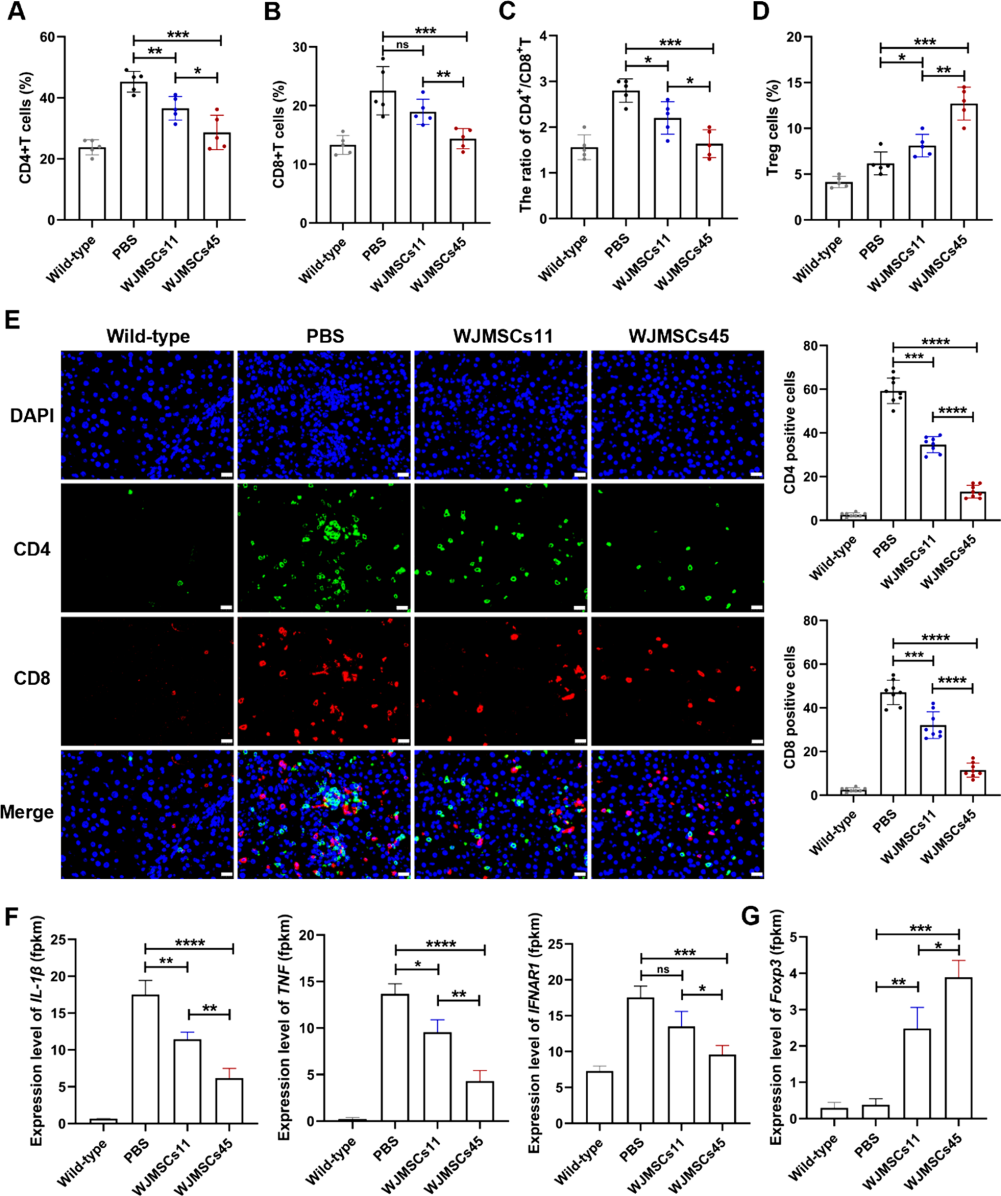
WJ-MSCs attenuate ConA-induced autoimmune hepatitis by regulating T and inflammation. **A**–**D** The quantification of T lymphocyte subsets (CD4^+^T, CD8^+^T, CD4/CD8 and Treg) in peripheral blood from mice were detected by flow cytometry. n = 5 in each group. **E** Quantification of CD4 or CD8-positive cells from liver in four group mice, wild-type, PBS-, WJ-MSCs11- and WJ-MSCs45- treatment after ConA injection. n = 6 in each group. Scale bar: 20 μm. **F**, **G** Transcriptome analysis of *IL-1β, TNF, IFNAR1* and *Foxp3* from liver in four group mice, wild-type, PBS, WJ-MSCs11 and WJ-MSCs45 treatment after ConA injection. n = 3 in each group. t tests were performed. Statistical significance is indicated by ^*^P < 0.05, ^**^P < 0.01, ^***^P < 0.001, ^****^P < 0.0001

To reveal the therapeutic mechanisms of WJ-MSCs for AIH, we analyzed the change of T lymphocyte subsets in peripheral blood and liver from mice. Flow cytometry revealed that treatment with WJ-MSCs significantly attenuated the proportion of CD4^+^T and CD8^+^T cells in PBMCs of mice with AIH (Fig. 5A, B, Additional file 1: Fig. S4A, B). Similarly, treatment with WJ-MSCs markedly decreased the CD4^+^T:CD8^+^T cell ratio and increased the number of Treg cells (Fig. 5C, D, Additional file 1: Fig. S4C). Immunostaining and quantitative analyses of CD4^+^T cells and CD8^+^T cells in liver were similar to the data for PBMCs (Fig. 5E, F). WJ-MSCs45 had a stronger immunoregulatory capacity than WJ-MSCs11. Consistent with phenotypes, transcriptome analysis showed that WJ-MSCs, especially PD-L1^high^ WJ-MSCs45, significantly inhibited the expression levels of genes encoding inflammatory factors, such as *IL-1β, TNF*, and *interferon-alpha/beta receptor subunit 1* (*IFNAR1*) in liver, but increased the expression of *Foxp3*, a transcription factor for Treg cells (Fig. 5 F, G). Together, these results indicated that PD-L1^high^ WJ-MSCs had stronger ability to alleviate ConA-induced AIH by inhibiting CD4^+^T and CD8^+^T cell proliferation, increasing Treg cell production, and reducing inflammation.

## Discussion

In this study, we reveal varieties of donor-derived WJ-MSCs in gene expression profiles and biological functions, including cell proliferation, differentiation bias, and immunoregulation. We found that individual heterogeneity among WJ-MSCs in immune regulation was positively correlated with the expression level of PD-L1 in WJ-MSCs. Importantly, exploration of the based on the treatment benefits of MSCs in a mouse model of ConA-induced AIH, we observed that the PD-L1^high^ WJ-MSCs had better therapeutic efficacy than PD-L1^low^ WJ-MSCs, which involved regulating the recipient’s T subset proliferation and inflammation. These findings implicated PD-L1 as a valuable indicator or biomarker to choose MSCs that produced effective clinical outcomes in treating autoimmune diseases.

Autoimmune hepatitis is a chronic liver disease characterized by immune-mediated destruction of hepatocytes [28]. Clinically, patients with AIH often manifest as elevated serum levels of ALT, AST, and immunoglobulin G, the presence of autoantibodies, and interface hepatitis [29]. In terms of pathogenic factors, the disease occurs in the absence of a clearly identified infectious agent but does appear to be triggered in some cases by drugs and other environmental factors [3]. Pathologically, many CD4^+^T cells and lymphocytic inflammatory infiltrate accompany variable hepatocyte necrosis and subsequent hepatic fibrosis [30]. Additionally, the levels of cytokines produced by CD4^+^T cells were significantly increased in serum of patients with AIH. In general, patients with AIH were treated with immunosuppressive agents, such as prednisone budesonide, mycophenolate mofetil, cyclosporine A, or tacrolimus [31]. Unfortunately, immunosuppressive therapy has wide-ranging side effects and results in infection and malignancy, which reduce patient quality of life and outcomes [32]. Moreover, Van den Brand FF et al. found that low doses of corticosteroids could still lead to substantial adverse events, such as bone fractures [33]. In most cases, when the drug is stopped and the disease can relapse, the effectiveness is lost [34]. Thus, alternatives to the traditional treatment for AIH patients are needed.

Currently, extensive studies have highlighted the broad potentials of MSCs in clinical applications. MSC-mediated immunoregulations have been confirmed in preclinical and clinical studies for many inflammatory and autoimmune diseases, including graft-versus-host disease (GvHD) [35], rheumatoid arthritis [36], systemic lupus erythematosus [37], inflammatory bowel disease [38], Sjogren’s syndrome [39], multiple sclerosis [40], type 1 diabetes mellitus [41], autoimmune liver disease [42] and other diseases. However, the therapeutic effects of MSCs in these clinical studies were quite varied and often not predictable. Although all MSCs from different tissues or donors share the general standards of MSCs proposed by the International Society for Cellular Therapy, the transcriptomics, proteomics, and epigenomics data are markedly heterogeneous. Several studies have compared gene expression similarities and variabilities among MSC samples [43–46]. These studies demonstrated that different gene expression profiles could reflect the ontogenetic sources of MSCs, differentiation potentials, and functional properties. In the present study, functional differences, including tri-lineage differentiation bias and immunomodulatory capability, were observed among different donor-derived WJ-MSCs using transcriptome analysis and in some cases specific gene expression levels. The findings provided a method to screen optimal MSCs in a disease-specific manner to ensure clinical benefits and reproductivity.

While increasing preclinical and clinical data support immunomodulatory properties of MSCs, there is still lack of a predictable therapeutic approach for MSCs-based therapies. Therefore, reliable potency assays are crucial to predict therapeutic effect of MSCs in the clinic. It is remarkable that MSCs from bone marrow for the treatment of acute GvHD are a good paradigm of this challenge. In this study, Kurtzberg J and his colleagues found that the expression of TNF receptor type 1 at a threshold level has been proposed as markers of both potency of MSCs [47]. Chinnadurai et al. discovered an approach to evaluate the immune modulatory potency of MSCs in vitro by combining molecular genetics and secretome analyses [48]. In the present study, PD-L1^high^ WJ-MSCs displayed better therapeutic efficacy for treating mice with AIH, suggesting that the level of PD-L1 expression may be an indicator of MSCs that will be beneficial in treating AIH patients.

Many studies have established that MSCs had significant immunoregulatory functions that regulated both innate and adaptive immune responses by direct or indirect cell-to-cell contact or paracrine mechanisms [49]. Various co-inhibitory surface molecules play an important role in immunosuppression by MSCs [50]. PD-L1 is a key immune molecule that mediates immune cells [51]. Evidence suggests that blocking PD-L1 signaling or knock-down of PD-L1 in MSCs results in the loss of the immunosuppressive function of T cells [11, 52]. We obtained similar results (Fig. 3D). Consistent with the finding that PD-L1 can promote both the induction and maintenance of induced Treg cells [53], our data showed that PD-L1^high^ WJ-MSCs increased the expression level of *Foxp3* and promoted the Treg cell production in vivo (Fig. 5D, G). Therefore, PD-L1 may inhibit T cell responses through promoting Treg cell production.

The present work provided some novel insights. To our knowledge, the study provided the first comprehensive gene expression profile of WJ-MSCs by analyzing large samples of different donor-derived WJ-MSCs at the transcriptome level. This will be an important data resource to understand the heterogeneity of WJ-MSCs. In addition, our study is the first to experimentally identify an indicator or marker (PD-L1) to screen WJ-MSCs that are optimal for treating AIH.

This study had some limitations. The detailed mechanisms of PD-L1-mediated immunoregulation of WJ-MSCs and the therapeutic mechanisms of PD-L1high WJ-MSCs on AIH need to be further explored. In addition, it is obscure whether other indicators or markers in combination with PD-L1 can be used to screen MSCs that more effectively treat AIH. Previous studies showed that ICAM1 is an important cell adhesion molecule, and its overexpression can enhance the immunomodulatory activity of MSCs by making MSCs more adhesive to T cells [54, 55]. Moreover, Zhang and his colleagues found that ICAM1-lymphocyte-function-associated antigen-1-mediated adhesion between tumor-derived extracellular vesicles, and T cells was a prerequisite for exosomal PD-L1-mediated immune suppression [56]. In the study, we also observed that ICAM1 was highly expressed in MSCs (the average of fpkm is 19.86). It is worth further exploring whether ICAM1 is a candidate marker for the immunomodulatory capacity of MSCs.

Overall, it is very important to develop specific and reliable potency assays of MSCs for diverse diseases, according to the disease pathogenesis, mechanism, and process. This strategy can eliminate variations in MSCs and improve therapeutic outcomes for MSCs-based cell therapy.

## Conclusion

We performed a comprehensive investigation of the heterogeneity of different donor-derived WJ-MSCs. The data implicated PD-L1 as an indicator to choose MSCs for the clinical treatment of AIH. These findings also provided novel insights into the quality control of MSCs and will lead to improvements in the therapeutic benefits of MSCs.

### Supplementary Information

**Additional file 1**. **Figure S1**. Characterization of WJ-MSCs isolated from 58 donors, relative to Figure 1. **Figure S2**. Analysis of single-cell RNA-sequencing data reveals intra-source variation of WJ-MSCs. **Figure S3**. Flow cytometry showing that different donor-derived WJ-MSCs with different expression levels of PD-L1 exhibited different functions on T cells, relative to Figure 3. **Figure S4**. Flow cytometry showing the changes of T lymphocyte subsets in peripheral blood from mice after different treatments, relative to Figure 5.

## Data Availability

All of RNA-seq data and scRNA-seq data were upload to Genome Sequence Archive for human database and were made public. the ID of RNA-seq data: OMIX003903, the ID of scRNA-seq data: HRA004510.

## Abbreviations

AILD: Autoimmune liver disease
AIH: Autoimmune hepatitis
ALT: Alanine aminotransferase
AST: Aspartate aminotransferase
ConA: Concanavalin A
GvHD: Graft-versus-host disease
IBD: Inflammatory bowel disease
MKSCs: Kiru mesenchymal stromal cells
MS: Multiple sclerosis
PD-L1: Programmed death-ligand 1
PBMCs: Peripheral blood mononuclear cells
RA: Rheumatoid arthritis
SLE: Systemic lupus erythematosus
SS: Sjogren’s syndrome
ISCT: International Society for Cellular Therapy
scRNA-seq: Single-cell RNA sequencing
T1DM: Type 1 diabetes mellitus
TNFR1: TNF receptor type 1
Tregs: Regulatory T cells
WJ-MSCs: Wharton's jelly derived mesenchymal stromal cells

## References

[1] Doherty DG (2016). Immunity, tolerance and autoimmunity in the liver: a comprehensive review. J Autoimmun.

[2] Lv TT, Li M, Zeng N (2019). Systematic review and meta-analysis on the incidence and prevalence of autoimmune hepatitis in Asian, European, and American population. J Gastroenterol Hepatol.

[3] Mieli-Vergani G, Vergani D, Czaja AJ (2018). Autoimmune hepatitis. Nat Rev Dis Primers.

[4] Richardson N, Ng STH, Wraith DC (2020). Antigen-specific immunotherapy for treatment of autoimmune liver diseases. Front Immunol.

[5] Mattner J (2011). Genetic susceptibility to autoimmune liver disease. World J Hepatol.

[6] Selvarajah V, Montano-Loza AJ, Czaja AJ (2012). Systematic review: managing suboptimal treatment responses in autoimmune hepatitis with conventional and nonstandard drugs. Aliment Pharmacol Ther.

[7] Ghannam S, Bouffi C, Djouad F (2010). Immunosuppression by mesenchymal stem cells: mechanisms and clinical applications. Stem Cell Res Ther.

[8] Krampera M, Le Blanc K (2021). Mesenchymal stromal cells: putative microenvironmental modulators become cell therapy. Cell Stem Cell.

[9] Zhang CX, Zhou LQ, Wang Z (2021). Eradication of specific donor-dependent variations of mesenchymal stem cells in immunomodulation to enhance therapeutic values. Cell Death Dis.

[10] Li YH, Zhang D, Xu L (2019). Cell-cell contact with proinflammatory macrophages enhances the immunotherapeutic effect of mesenchymal stem cells in two abortion models. Cell Mol Immunol.

[11] Zhou KJ, Guo S, Tong S (2018). Immunosuppression of human adipose-derived stem cells on T cell subsets via the reduction of NF-kappaB activation mediated by PD-L1/PD-1 and Gal-9/TIM-3 pathways. Stem Cells Dev.

[12] Ma SF, Chen XH, Wang LH (2017). Repairing effects of ICAM-1-expressing mesenchymal stem cells in mice with autoimmune thyroiditis. Exp Ther Med.

[13] Zhang CY, Zhu Y, Wang J (2019). CXCR4-overexpressing umbilical cord mesenchymal stem cells enhance protection against radiation-induced lung injury. Stem Cells Int.

[14] He JG, Li BB, Zhou L (2020). Indoleamine 2,3-dioxgenasetransfected mesenchymal stem cells suppress heart allograft rejection by increasing the production and activity of dendritic cells and regulatory T cells. J Investig Med.

[15] Zhang Z, Huang S, Wu S (2019). Clearance of apoptotic cells by mesenchymal stem cells contributes to immunosuppression via PGE2. EBioMedicine.

[16] Davies LC, Heldring N, Kadri N (2017). Mesenchymal stromal cell secretion of programmed death-1 ligands regulates T cell mediated immunosuppression. Stem Cells.

[17] Bulati M, Miceli V, Gallo A (2020). The immunomodulatory properties of the human amnion-derived mesenchymal stromal/stem cells are induced by INF-gamma produced by activated lymphomonocytes and are mediated by cell-to-cell contact and soluble factors. Front Immunol.

[18] Camilleri ET, Gustafson MP, Dudakovic A (2016). Identification and validation of multiple cell surface markers of clinical-grade adipose-derived mesenchymal stromal cells as novel release criteria for good manufacturing practice-compliant production. Stem Cell Res Ther.

[19] Volarevic V, Mitrovic M, Milovanovic M (2012). Protective role of IL-33/ST2 axis in Con A-induced hepatitis. J Hepatol.

[20] Liu L, Wei Q, Lin QQ (2019). Anti-spike IgG causes severe acute lung injury by skewing macrophage responses during acute SARS-CoV infection. JCI Insight.

[21] Pabla S, Conroy JM, Nesline MK (2019). Proliferative potential and resistance to immune checkpoint blockade in lung cancer patients. J Immunother Cancer.

[22] Crisan M, Yap S, Casteilla L (2008). A perivascular origin for mesenchymal stem cells in multiple human organs. Cell Stem Cell.

[23] Mendez-Ferrer S, Michurina TV, Ferraro F (2010). Mesenchymal and haematopoietic stem cells form a unique bone marrow niche. Nature.

[24] Yu Y, Deng P, Yu B (2017). Inhibition of EZH2 promotes human embryonic stem cell differentiation into mesoderm by reducing H3K27me3. Stem Cell Rep.

[25] Weatherall EL, Avilkina V, Cortes-Araya Y (2020). Differentiation potential of mesenchymal stem/stromal cells is altered by intrauterine growth restriction. Front Vet Sci.

[26] Sarjeant K, Stephens JM (2012). Adipogenesis. Cold Spring Harb Perspect Biol.

[27] So CL, Kaluarachchi K, Tam PP (2001). Impact of mutations of cartilage matrix genes on matrix structure, gene activity and chondrogenesis. Osteoarthr Cartil.

[28] Mieli-Vergani G, Vergani D (2011). Autoimmune hepatitis. Nat Rev Gastroenterol Hepatol.

[29] Liberal R, Grant CR, Mieli-Vergani G (2013). Autoimmune hepatitis: a comprehensive review. J Autoimmun.

[30] Webb GJ, Hirschfield GM, Krawitt EL (2018). Cellular and molecular mechanisms of autoimmune hepatitis. Annu Rev Pathol.

[31] Corrigan M, Hirschfield GM, Oo YH (2015). Autoimmune hepatitis: an approach to disease understanding and management. Br Med Bull.

[32] Harrington C, Krishnan S, Mack CL (2022). Noninvasive biomarkers for the diagnosis and management of autoimmune hepatitis. Hepatology.

[33] Van den Brand FF, Van der Veen KS, Lissenberg-Witte BI (2019). Adverse events related to low dose corticosteroids in autoimmune hepatitis. Aliment Pharmacol Ther.

[34] Montano-Loza AJ, Carpenter HA, Czaja AJ (2007). Consequences of treatment withdrawal in type 1 autoimmune hepatitis. Liver Int.

[35] Boberg E, von Bahr L, Afram G (2020). Treatment of chronic GvHD with mesenchymal stromal cells induces durable responses: a phase II study. Stem Cells Transl Med.

[36] Ghoryani M, Shariati-Sarabi Z, Tavakkol-Afshari J (2019). Amelioration of clinical symptoms of patients with refractory rheumatoid arthritis following treatment with autologous bone marrow-derived mesenchymal stem cells: a successful clinical trial in Iran. Biomed Pharmacother.

[37] Deng DQ, Zhang PL, Guo Y (2017). A randomised double-blind, placebo-controlled trial of allogeneic umbilical cord-derived mesenchymal stem cell for lupus nephritis. Ann Rheum Dis.

[38] Panés J, García-Olmo D, Van Assche G (2016). Expanded allogeneic adipose-derived mesenchymal stem cells (Cx601) for complex perianal fistulas in Crohn’s disease: a phase 3 randomised, double-blind controlled trial. Lancet.

[39] Xu JJ, Wang DD, Liu DY (2012). Allogeneic mesenchymal stem cell treatment alleviates experimental and clinical Sjögren syndrome. Blood.

[40] Petrou P, Kassis I, Levin N (2020). Beneficial effects of autologous mesenchymal stem cell transplantation in active progressive multiple sclerosis. Brain.

[41] Lu J, Shen SM, Ling Q (2021). One repeated transplantation of allogeneic umbilical cord mesenchymal stromal cells in type 1 diabetes: an open parallel controlled clinical study. Stem Cell Res Ther.

[42] Wang LF, Li J, Liu HH (2013). Pilot study of umbilical cord-derived mesenchymal stem cell transfusion in patients with primary biliary cirrhosis. J Gastroenterol Hepatol.

[43] Jansen BJ, Gilissen C, Roelofs H (2010). Functional differences between mesenchymal stem cell populations are reflected by their transcriptome. Stem Cells Dev.

[44] Wagner W, Wein F, Seckinger A (2005). Comparative characteristics of mesenchymal stem cells from human bone marrow, adipose tissue, and umbilical cord blood. Exp Hematol.

[45] Cho KA, Park M, Kim YH (2017). RNA sequencing reveals a transcriptomic portrait of human mesenchymal stem cells from bone marrow, adipose tissue, and palatine tonsils. Sci Rep.

[46] Roson-Burgo B, Sanchez-Guijo F, Del Canizo C (2014). Transcriptomic portrait of human mesenchymal stromal/stem cells isolated from bone marrow and placenta. BMC Genomics.

[47] Kurtzberg J, Prockop S, Teira P (2014). Allogeneic human mesenchymal stem cell therapy (remestemcel-L, Prochymal) as a rescue agent for severe refractory acute graft-versus-host disease in pediatric patients. Biol Blood Marrow Transplant.

[48] Chinnadurai R, Rajan D, Qayed M (2018). Potency analysis of mesenchymal stromal cells using a combinatorial assay matrix approach. Cell Rep.

[49] Shi YF, Wang Y, Li Q (2018). Immunoregulatory mechanisms of mesenchymal stem and stromal cells in inflammatory diseases. Nat Rev Nephrol.

[50] Takizawa N, Okubo N, Kamo M (2017). Bone marrow-derived mesenchymal stem cells propagate immunosuppressive/anti-inflammatory macrophages in cell-to-cell contact-independent and-dependent manners under hypoxic culture. Exp Cell Res.

[51] Keir ME, Butte MJ, Freeman GJ (2008). PD-1 and its ligands in tolerance and immunity. Annu Rev Immunol.

[52] Wu WJ, Lan Q, Lu H (2014). Human amnion mesenchymal cells negative co-stimulatory molecules PD-L1 expression and its capacity of modulating microglial activation of CNS. Cell Biochem Biophys.

[53] Francisco LM, Salinas VH, Brown KE (2009). PD-L1 regulates the development, maintenance, and function of induced regulatory T cells. J Exp Med.

[54] Hua SS (2013). Targeting sites of inflammation: intercellular adhesion molecule-1 as a target for novel inflammatory therapies. Front Pharmacol.

[55] Tang B, Li X, Liu YL (2018). The therapeutic effect of ICAM-1-overexpressing mesenchymal stem cells on acute graft-versus-host disease. Cell Physiol Biochem.

[56] Zhang W, Zhong WQ, Wang BK (2022). ICAM-1-mediated adhesion is a prerequisite for exosome-induced T cell suppression. Dev Cell.

