# Supplementary material for: PD-L1 expression levels in mesenchymal stromal cells predict their therapeutic values for autoimmune hepatitis
Source: Stem Cell Res Ther. 2023 Dec 18;14:370. doi: 10.1186/s13287-023-03594-z (PMC10729378; doi:10.1186/s13287-023-03594-z)
Supplement: Supplementary file 1 — Additional file 1. Figure S1. Characterization of WJ-MSCs isolated from 58 donors, relative to Figure 1. Figure S2. Analysis of single-cell RNA-sequencing data reveals intra-source variation of WJ-MSCs. Figure S3. Flow cytometry showing that different donor-derived WJ-MSCs with different expression levels of PD-L1 exhibited different functions on T cells, relative to Figure 3. Figure S4. Flow cytometry showing the changes of T lymphocyte subsets in peripheral blood from mice after different treatments, relative to Figure 5. [file 13287_2023_3594_MOESM1_ESM.docx]

**Supplementary information**

**
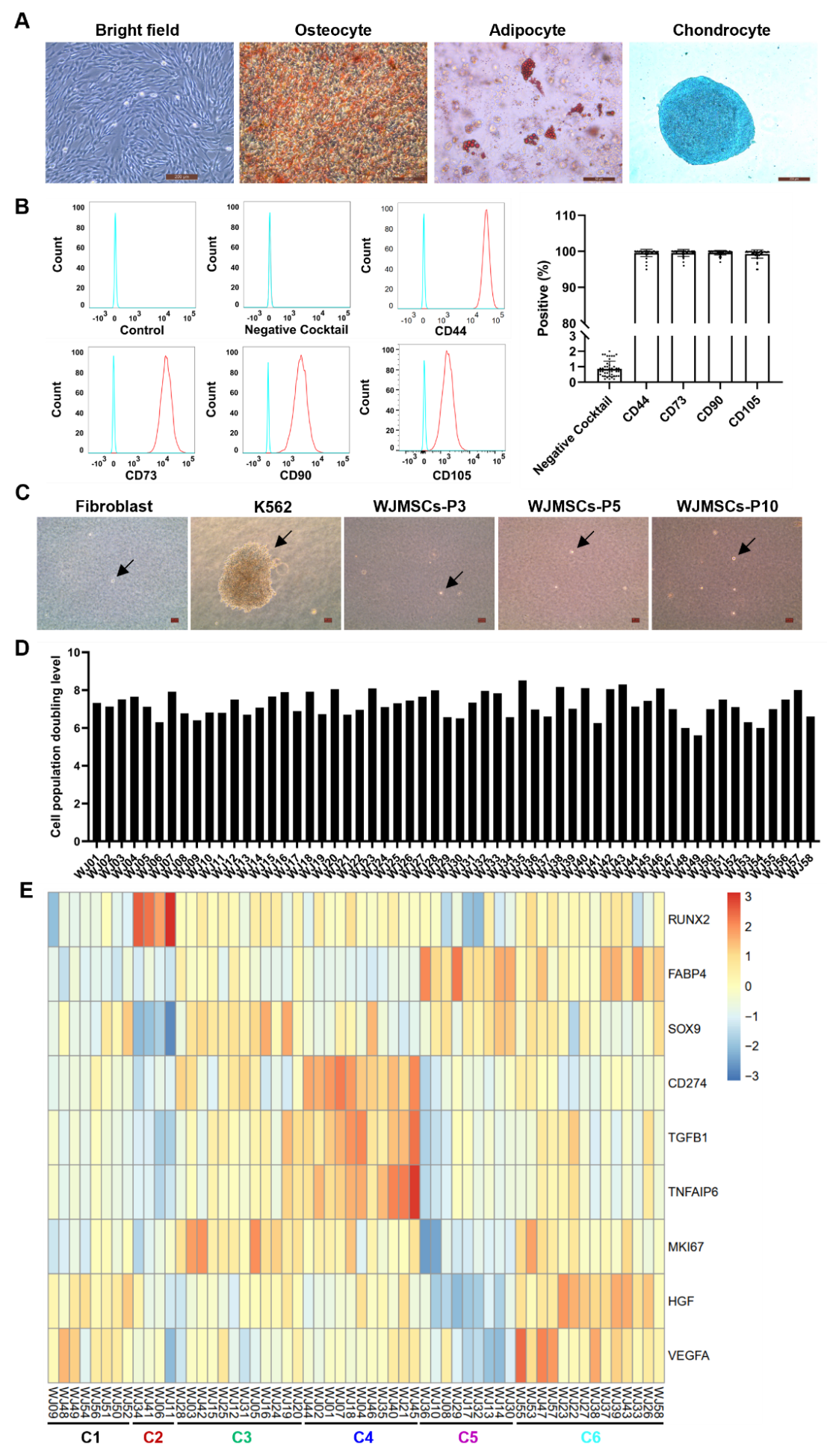
**

**Figure S1. Characterization of WJ-MSCs isolated from 58 donors, relative to Figure 1.** A. Reprensentaitve images of WJ-MSCs after in vitro differentiation into adipocytes (oil red O), chondrocytes (alcian blue) and osteocytes (alizarin red). B. Flow cytometry assays showed WJ-MSCs were negative for CD14, CD19, CD34, CD45, and HLA-DR, but positive for CD44, CD73, CD90, and CD105. C. Soft agar clone formation assays showing clone formation abilities of WJ-MSCs at passage 3, 5 and 10. K562 cell lines and normal fibroblasts (HFF-1) were used as positive reference and negative reference, respectively. D. Comparsion of the cell population doubling time of 58 WJ-MSCs at P3(P1-P3). E. Heat map of the representiave genes across 58 different donor-derived MSCs.

**
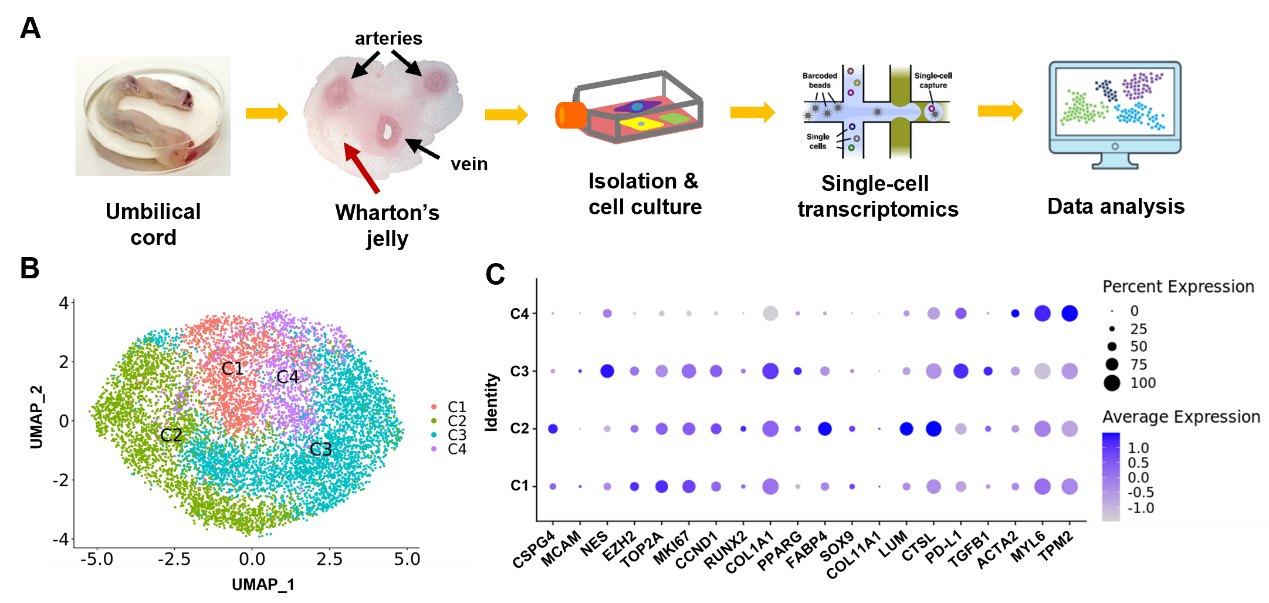
**

**Figure S2. Analysis of single-cell RNA-sequencing data reveals intra-source variation of WJ-MSCs.** A. Flow chart of sc-RNAseq analysis of WJ-MSCs. B. UMAP plots showing subpopulation in WJ-MSCs. C. Bubble diagram depicting the gene expressions of special genes in four subpopulations of MSCs, including perivascular mesodermal progenitor cell markers (*CSPG4, MCAM, NES* and *EZH2*)*,* proliferation markers (*TOP2A, MKI67* and *CCND1*)*,* trilineage differentiation markers (*RUNX2, COL1A1, PPARG, FABP4, SOX9* and *COL11A1*)*,* extracellular matrix remodeling markers (*LUM* and *CTSL*), immunoregulatory markers (*PD-L1* and *TGFB1*), smooth muscle contraction smooth muscle contraction (*ACTA2*, *MYL6* and *TPM2*).


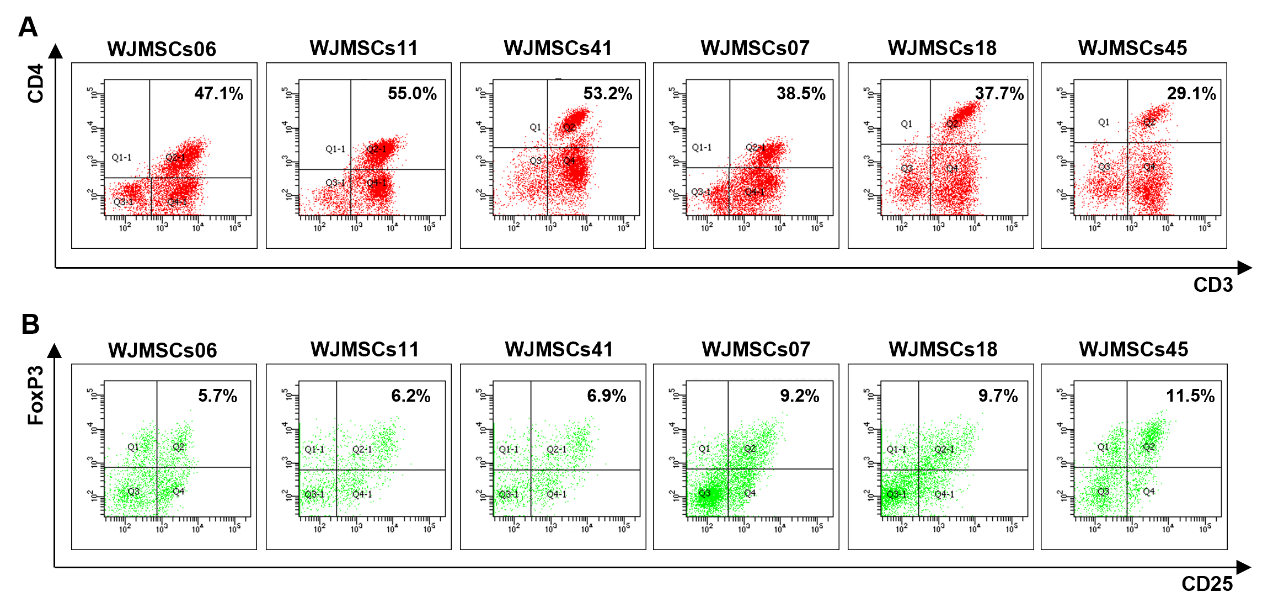


**Figure S3.** **Flow cytometry showing that different donor-derived WJ-MSCs with different expression levels of PD-L1 exhibited different functions on T cells, relaitve to Figure 3.** A-B. The effects of WJ-MSCs in inhibiting the proportion of CD4^+^T cells and increasing the production of Tregs subpopulation in PBMCs were detected by flow cytometry. n=3 in each group.

**
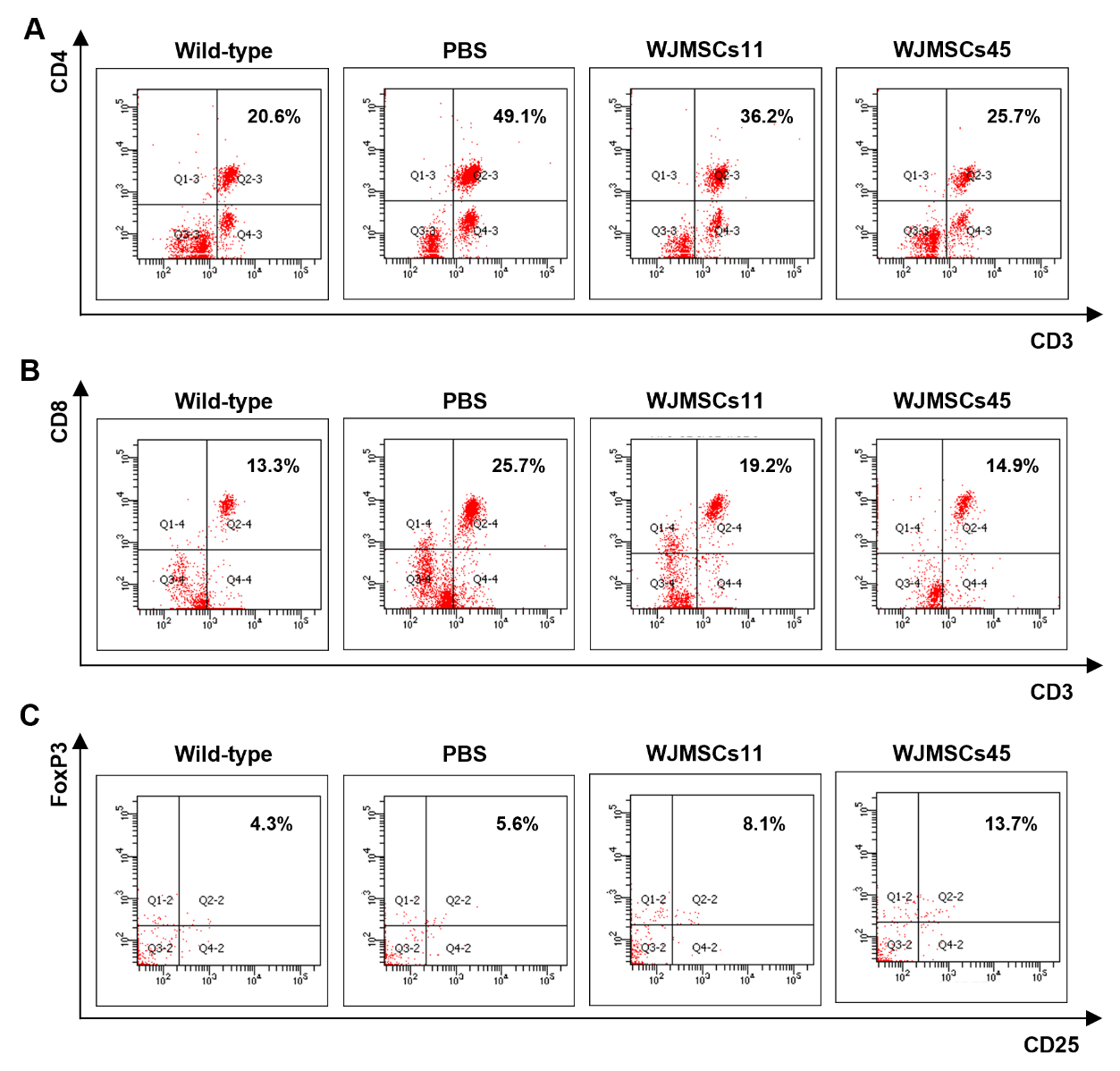
**

**Figure S4. Flow cytometry showing the changes of T lymphocyte subsets (CD4**^+^**T, CD8**^+^**T, Treg) in peripheral blood from mice after different treatments, relative to Figure 5.** A-C. The quantification of T lymphocyte subsets (CD4^+^T, CD8^+^T and Treg) in peripheral blood from mice were detected by flow cytometry. n=5 in each group.
